# Sleep Disturbances Are Associated With Depressive Symptoms in a Chinese Population: The Rugao Longevity and Aging Cohort

**DOI:** 10.3389/fpsyt.2021.731371

**Published:** 2021-12-08

**Authors:** Chunhong Qiao, Hui Zhang, Qi Song, Xi Wang, Xiaofeng Wang, Yin Yao

**Affiliations:** ^1^Department of Biostatistics and Computational Biology, School of Life Sciences, Fudan University, Shanghai, China; ^2^Human Phenome Institute, Fudan University, Shanghai, China; ^3^State Key Laboratory of Genetic Engineering and Ministry of Education (MOE) Key Laboratory of Contemporary Anthropology, School of Life Sciences, Fudan University, Shanghai, China

**Keywords:** sleep disturbance, depressive symptoms, Chinese older population, risk factor, sleep efficiency

## Abstract

**Objective:** To investigate the cross-sectional and longitudinal relationships between sleep disturbances and depressive symptoms in older Chinese adults.

**Methods:** This study included baseline and 3.5-year follow-up data of 1,631 Chinese men and women aged 70 years or older from the aging arm of the Rugao Longevity and Aging Study. Depressive symptoms were assessed by the 15-item Geriatric Depression Scale (GDS). Sleep disturbances were assessed by using the Pittsburgh Sleep Quality Index (PSQI). Logistic regression models were used to estimate the odds ratios (ORs) of the associations.

**Results:** In the cross-sectional analysis, individuals with greater total PSQI scores exhibited significantly higher risk of “depressive symptoms” (OR: 1.31, 95% CI: 1.21–1.41) and “some depressive symptoms” (OR: 1.22, 95% CI: 1.17–1.28). Specifically, higher scores on the sleep efficiency PSQI subscale were associated with greater odds for “depressive symptoms” (OR: 1.54, 95% CI: 1.30–1.84) and “some depressive symptoms” (OR: 1.42, 95% CI: 1.29–1.57). Our longitudinal analyses indicated an association between greater PSQI total scores at baseline and greater odds of having “some depressive symptoms” at follow-up (OR: 1.07, 95% CI: 1.00–1.14). Additionally, higher scores on the sleep efficiency PSQI subscale had an association with higher odds for “some depressive symptoms” (OR: 1.21, 95% CI: 1.04–1.41).

**Conclusions:** Poor self-reported global sleep quality and sleep efficiency PSQI subscale scores were associated with levels of depressive symptoms in an older Chinese population, indicating that global sleep quality and sleep efficiency may be risk factors for depression and can possibly predict the levels of depressive symptoms.

## Introduction

Depression among the elderly population, with an estimated prevalence of 8–16%, is a major public health problem that has attracted worldwide attention (1, 2). Depression presents a heavy disease burden of long-term care on families and society as a whole (3, 4). Therefore, the prevention and treatment of depression have become urgent tasks in the field of public health. Depression has been recognized as being associated with genetic (5), physical, behavioral, and socioeconomic factors (6). A potential effort to reduce depression levels has been targeting sleep disturbances.

Two previous longitudinal studies revealed that poor self-reported sleep quality was associated with an increased risk of depression. One analysis was conducted in the context of the Study of Osteoporotic Fractures (SOF), and the other was conducted within the prospective Osteoporotic Fractures in Men (MrOS) study. Both studies utilized the Geriatric Depression Scale (GDS) to measure depressive symptoms. Maglione et al. reported that baseline sleep disturbances were associated with a greater chance of worse depressive symptoms 5 years later (7). Paudel et al. revealed that among non-depressed older men, poor self-reported sleep quality was also associated with increased odds of depression 3.4 years later (8).

Before sleep disturbance can be established as a risk factor for depression, more evidence needs to be accumulated in different ethnic and age groups within older populations. In this study, we aimed to explore the relationship between sleep disturbances and depression symptoms at baseline in 1,631 Chinese adult participants aged 70 years or older. We also reassessed depressive symptoms after a 3.5-year follow up, further probing into the question of whether sleep disturbances or its subcomponents at baseline could potentially predict future depressive symptoms.

## Methods

### Participants

**Figure 1:**
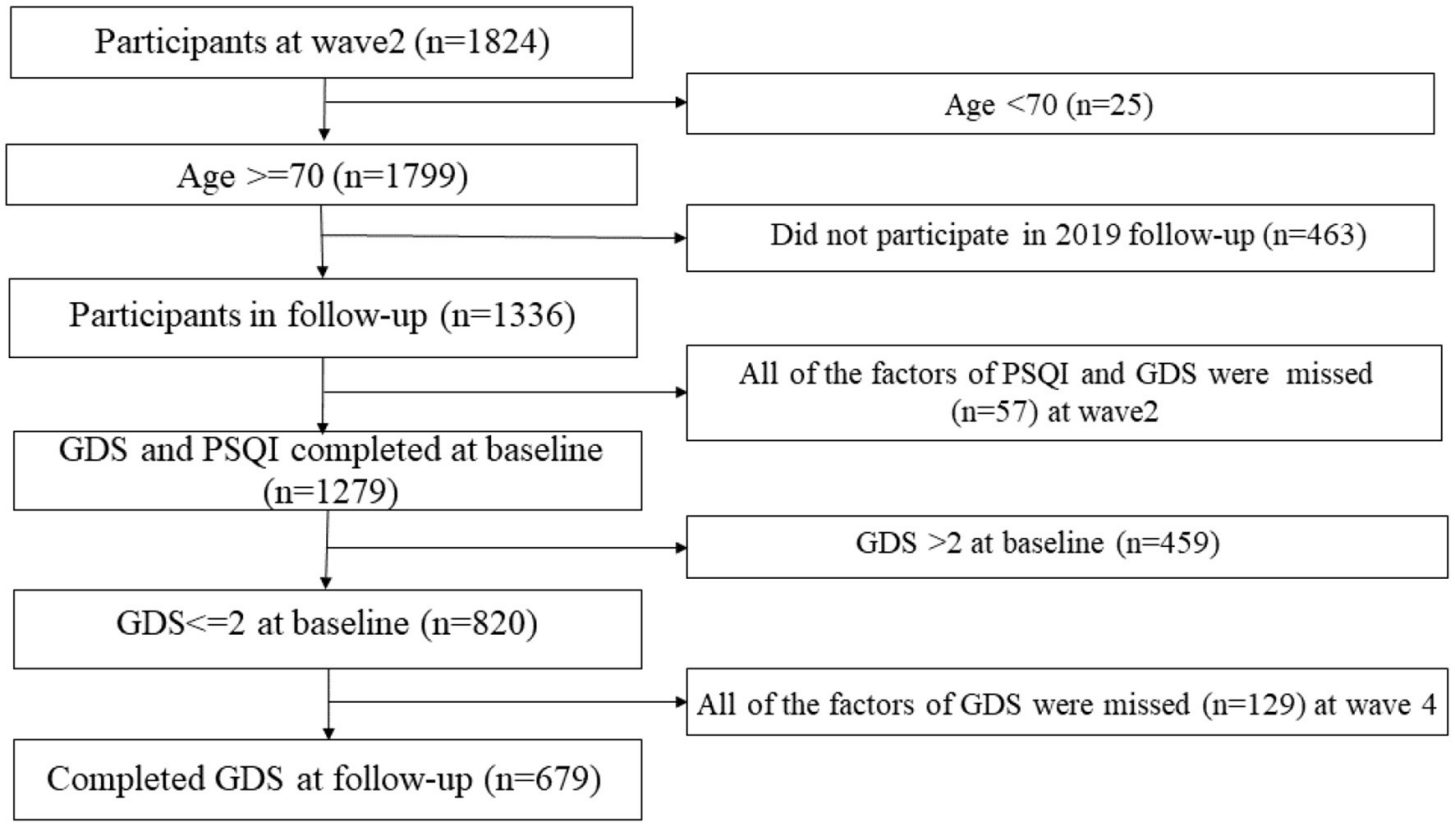
Recruitment and inclusion of participants. Reasons for not including participants are given on the right. GDS, Geriatric Depression Scale; *n*, number; RuLAS, Rugao Longevity and Aging Study.

The data came from the aging arm of the Rugao Longevity and Aging Study (RuLAS), a population-based, observational, two-arm cohort study conducted in Rugao, Jiangsu Province, China. Approximately 1960 older adults were recruited based on 5-year age and sex strata, equally among 31 villages, in 2014. Our first follow-up was conducted after 1.5 years in the summer of 2016, and the second follow-up was conducted in the winter of 2017 (3 years after baseline). The third follow-up was conducted in the winter of 2019 (5 years after baseline) (9, 10). The current study focused on participants in the second and fourth waves (~1.5 and 5 years after the original assessment) of the RuLAS. In this study, the second wave was recognized as the baseline because data from the Pittsburgh Sleep Quality Index (PSQI) questionnaire were collected starting with the second wave. In the cross-sectional analysis, a total of 1,631 participants were included after excluding participants who appeared to suffer from major diseases (stroke, myocardial infarction, and cancer). Out of 1,631 individuals, 1,279 participants returned and completed both GDS and PSQI questionnaires at baseline. After excluding 459 participants who reported “some depressive symptoms” (GDS 3–5) or “depressive symptoms” (GDS ≥6), the remaining 820 participants had reported few depressive symptoms (GDS 0–2). Of these, including older adults with few depressive symptoms, 679 completed GDS questionnaires at the 3-year follow-up. Our longitudinal analyses were conducted on this subset of 679 participants. A schematic of the inclusion and exclusion of older Chinese adults in the longitudinal analysis is shown in Figure 1. The Human Ethics Committee of the School of Life Sciences of Fudan University, Shanghai, China, approved this study (No: BE1815). Written informed consent was obtained from all participants prior to the study.

### Depressive Symptoms

Depressive symptoms were assessed using the Chinese version of the 15-item GDS (11). This form of the GDS consisted of 15 self-reported yes-or-no questions that were derived from the GDS-30. The GDS-15 scale has previously been validated for use in community-living older Asian adults (12). A previous review reported that the best performance for the GDS was with a cutoff of 5/6 for the GDS-15 (13). A standard cutoff of ≥ 6 on the GDS revealed 91% sensitivity and 65% specificity when evaluated against diagnostic criteria (14). In this study, we implemented the validated cutoff of 6 to capture no/some and high depressive symptoms. Depressive symptoms were categorized into three groups, based on clinical relevance [0–2 (normal), 3–5 (some depressive symptoms) and ≥ 6 (depressed)] (7). For simplicity and consistency, patients with particular levels of depressive symptoms at follow-up were divided into three groups [0–2 (normal), 3–5 (some depressive symptoms) and ≥ 6 (depressed)]. The changes in GDS score between baseline and follow-up were calculated for each study participant. The participants with an increased GDS score ≥ 2 points were considered to have “worsening depressive symptoms.” This threshold was chosen based upon the distribution of changes in GDS scores in our samples because the GDS scores used in this study were able to distinguish older Chinese adults with the greatest increase in GDS score (highest quartile) between baseline and 3-year follow-up (7).

### Sleep Measures

Global sleep quality was assessed using the PSQI (15). The PSQI is a validated 19-item self-reported measure of sleep disturbances. It has been validated and demonstrated to have good psychometric properties in an ethnically similar population (16). The Chinese version of the PSQI has good overall reliability (*r* = 0.82–0.83) and test-retest reliability (*r* = 0.77–0.85) in community of adults with primary insomnia (16, 17). The PSQI had internal consistency and a reliability coefficient (Cronbach's alpha) of 0.703 for its seven components. The PSQI was divided into seven subcomponent scores: sleep quality, sleep latency, sleep duration, sleep efficiency, sleep disturbance, sleep medication use, and daytime dysfunction. Each subcomponent score ranges from 0 to 3, and global PSQI scores range from 0 to 21, with higher scores reflecting more severe symptoms. Total PSQI scores are expressed as a continuous variable and as a categorical variable: PSQI > 5 vs. PSQI ≤ 5 (18). Questionnaires were administered by trained physicians. The trained physicians were present during the entire duration of the assessment to provide clarifications if participants had trouble understanding any of the items in the questionnaires. These measures were verbally administered by these physicians in cases where the participants were illiterate.

### Covariables

Demographic information included age, sex (male or female), marital status (currently married, others), and education (illiterate, literate). Lifestyle included smoking status (non-smoker, smoker), drinking status (non-drinker, drinker), body mass index (BMI) [normal or underweight (<24.0), overweight (24.0–27.9), and obese (≥28.0)] and self-reported health status.

### Statistical Analyses

Continuous variables are reported as the mean ± standard deviation (SD), and categorical variables are reported as percentages. The relationship between sleep disturbance and depressive symptoms were assessed using logistic regression models. The differences in the characteristics of the participants at baseline and at the 3.5-year follow-up based on the level of depressive symptoms were also assessed using logistic regression models. Covariates known to be associated with levels of depressive symptoms at follow-up or with sleep disturbances were included in multivariable models. Logistic regression models were first used to estimate the odds ratio (ORs) for falling into different depressive symptom level categories (i.e., “some depressive symptoms” (GDS 3–5) or “depressed” (GDS ≥ 6) at follow-up). Logistic regression models were then used to estimate the OR for having a two-point or greater increase in GDS score at follow-up. Models were adjusted for age, sex, education, marriage status, smoking status, drinking status and self-reported health status for multiple variables. *P*-values < 0.05 were considered statistically significant. All analyses were conducted using R x64 4.0.2 (“https://www.r-project.org/,”).

## Results

### Characteristics of the Participants

Table 1 describes the baseline characteristics of the study population categorized into different depressive symptom groups. The mean age was 77.2 ± 4.12 years; 51.44% (*n* = 839) were females; 47.03% (*n* = 767) were illiterate; and 63.83% (*n* = 1041) were currently married. A total of 6.81% (*n* = 111) of the participants had depressive symptoms, and 16.19% (*n* = 264) had sleep disturbance at baseline. Smoking status and drinking status were associated with “some depressive symptoms” and “no or few depressive symptoms.” There was no significant difference among the groups with “depressive symptoms,” “some depressive symptoms,” and “no or few depressive symptoms” in age, sex, marital status, literacy, or BMI. Of the participants without evidence of depression at baseline, 31.66% exhibited “some depressive symptoms” and 6.48% revealed “depressive symptoms” at the 3.5-year follow-up examination.

**Table 1:** Characteristics of participants according to the level of depressive symptoms at baseline.

**Characteristics**	**All** **(*n* = 1,631)**	**GDS ≥ 6** **(*n* = 111)**	**3 ≤ GDS ≤ 5** **(*n* = 489)**	**0 ≤ GDS ≤ 2** **(*n* = 1,031)**	***P*-value**
**Age group**, ***n*** **(%)**					0.372
70–74	481 (29.49)	41 (36.94)	128 (26.18)	312 (30.03)	
75–79	687 (42.12)	41 (36.94)	200 (40.90)	446 (42.93)	
80–84	368 (22.56)	18 (16.22)	128 (26.18)	222 (21.37)	
85+	95 (5.82)	11 (9.91)	33(6.75)	51(4.91)	
**Sex**					0.093
Male	787 (48.25)	58 (52.25)	217 (44.38)	512 (49.28)	
Female	839 (51.44)	53 (47.75)	272 (55.62)	514 (49.47)	
**Education**					0.597
Illiterate	767 (47.03)	56 (50.45)	258 (52.76)	515 (49.57)	
Literate	829 (50.83)	55 (49.55)	221 (45.19)	491 (47.26)	
**Marital status**					0.396
Current marital	1,041 (63.83)	70 (63.06)	305 (62.37)	666 (64.10)	
Other	547 (33.54)	41 (36.94)	175 (35.79)	331 (31.86)	
**Smoking**					**<0.001**
None	1,425 (87.37)	76 (68.47)	422 (86.30)	902 (86.81)	
Smoker	132 (8.09)	32 (28.83)	46 (9.41)	79 (7.60)	
**Drinking**					**<0.001**
None	1,412 (86.57)	62 (55.86)	425 (86.91)	888 (85.47)	
Drinker	134 (8.22)	45 (40.54)	41 (8.38)	85 (8.18)	
**BMI category**					0.067
<24	907 (55.61)	62 (55.86)	259 (52.97)	586 (56.40)	
24–28	533 (32.68)	43 (38.74)	173 (35.38)	317 (30.51)	
≥28	175 (10.73)	5 (4.50)	53 (10.84)	117 (11.26)	
**PSQI score**					
≤5	952 (58.37)	43 (38.74)	194 (39.67)	715 (69.35)	
>5	679 (41.63)	68 (61.26)	295 (60.33)	316 (30.65)	

*Others in marital status means windowed, divorced, and unmarried. GDS, Geriatric Depression Scale; PSQI, Pittsburgh Sleep Quality Index; SD, standard deviation; BMI, body mass index; Bold means statistically significant*.

### Associations Between Sleep Disturbances and Depressive Symptoms at Baseline

Table 2 indicates that poor global sleep quality (PSQI > 5) was significantly associated with the follow up “some depressive symptoms” (OR: 3.44, 95% CI: 2.75–4.31) and “depressive symptoms” (OR: 3.58, 95% CI: 2.40–5.40) after adjusting for confounding factors. PSQI subcomponents, including sleep quality (OR: 1.61, 95% CI: 1.10–2.34), sleep latency (OR: 1.67, 95% CI: 1.33–2.10), sleep duration (OR: 1.96, 95% CI: 1.48–2.59), sleep efficiency (OR: 1.54, 95% CI: 1.30–1.84), sleep disturbance (OR: 1.61, 95% CI: 1.03–2.49), and daytime dysfunction (OR: 1.62, 95% CI: 1.31–2.01), were also associated with “depressive symptoms” after adjusting for confounding factors.

**Table 2:** Associations between sleep disturbance and depressive symptom levels at baseline.

**Sleep variables**	**Some depressive symptoms** **OR (95% CI)**	***P*-value**	**Depressive symptoms** **OR (95% CI)**	***P*-value**
**PSQI Total Score**				
Base model	1.22 (1.17–1.27)	**<0.001**	1.30 (1.21–1.40)	**<0.001**
Multivariable adjusted	1.22 (1.17–1.28)	**<0.001**	1.31 (1.21–1.41)	**<0.001**
**PSQI >5 PSQI ≤5**				
Base model	3.44 (2.75–4.31)	**<0.001**	3.58 (2.40–5.40)	**<0.001**
Multivariable adjusted	3.56 (2.77–4.58)	**<0.001**	3.81 (2.45–6.02)	**<0.001**
**Sleep quality factor**				
Base model	1.37 (1.15–1.64)	**<0.001**	1.69 (1.23–2.33)	**0.001**
Multivariable adjusted	1.37 (1.10–1.70)	**0.004**	1.61 (1.10–2.34)	**0.013**
**Sleep latency factor**				
Base model	1.40 (1.24–1.59)	**<0.001**	1.69 (1.35–2.10)	**<0.001**
Multivariable adjusted	1.35 (1.18–1.54)	**<0.001**	1.67 (1.33–2.10)	**<0.001**
**Sleep duration factor**				
Base model	1.60 (1.35–1.89)	**<0.001**	1.91 (1.47–2.47)	**<0.001**
Multivariable adjusted	1.65 (1.38–1.97)	**<0.001**	1.96 (1.48–2.59)	**<0.001**
**Sleep efficiency factor**				
Base model	1.43 (1.31–1.57)	**<0.001**	1.55 (1.32–1.82)	**<0.001**
Multivariable adjusted	1.42 (1.29–1.57)	**<0.001**	1.54 (1.30–1.84)	**<0.001**
**Sleep disturbances factor**				
Base model	1.56 (1.24–1.97)	**<0.001**	1.76 (1.16–2.62)	**0.007**
Multivariable adjusted	1.46 (1.14–1.87)	**0.003**	1.61 (1.03–2.49)	**0.034**
**Sleep medication use factor**				
Base model	1.95 (1.16–3.65)	**0.018**	1.01 (0.07–3.24)	0.995
Multivariable adjusted	2.23 (1.16–5.19)	**0.029**	1.36 (0.09–6.45)	0.74
**Daytime dysfunction factor**				
Base model	1.35 (1.19–1.53)	**<0.001**	1.63 (1.33–2.00)	**<0.001**
Multivariable adjusted	1.33 (1.16–1.52)	**<0.001**	1.62 (1.31–2.01)	**<0.001**

*Odds ratios and 95% confidence intervals for falling into the “some depressive symptoms” or “depressed” groups are given according to baseline subjective sleep measures. GDS, Geriatric Depression Scale; PSQI, Pittsburgh Sleep Quality Index; CI, confidence interval. The base model p-value adjusted nothing, and the multivariable adjusted p-value adjusted for age, sex, education, marriage, drinking, smoking, and self-reported health status. Bold means statistically significant*.

### Associations Between Baseline Sleep Disturbances and Depressive Symptoms at Follow-Up

Table 3 indicates that global sleep quality was associated with “some depressive symptoms” (OR: 1.09, 95% CI: 1.03–1.15) at follow-up, and this association remained significant after adjusting for confounding factors. Poor global sleep quality (PSQI > 5) was associated with “some depressive symptoms” (OR: 1.44, 95% CI: 1.00–2.02), and no significant association was detected after adjusting for confounding factors. Interestingly, the PSQI subcomponents sleep latency (OR: 1.25, 95% CI: 1.03–1.51), sleep efficiency (OR: 1.25, 95% CI: 1.09–1.44), and sleep disturbance (OR: 1.25, 95% CI: 1.00–2.07) were associated with “some depressive symptoms.” After adjustment, only sleep efficiency retained an association with “some depressive symptoms” (OR: 1.21, 95% CI: 1.04–1.41). The category defined by PSQI score > 5 was associated with “depressive symptoms” (OR: 2.03, 95% CI: 1.09–3.82), while no association was revealed after adjusting cofounding factors. The PSQI subcomponent sleep efficiency had an association with “depressive symptoms” (OR: 1.34, 95% CI: 1.04–1.72). Interestingly, there was a lack of association between sleep efficiency and depressive symptoms after adjusting for confounding factors (OR: 1.21, 95% CI: 0.91–1.61). The PSQI subcomponents sleep quality, sleep duration, sleep medication use, and daytime dysfunction had no association with “some depressive symptoms” and “depressive symptoms.”

**Table 3:** Associations between sleep disturbance at baseline and depressive symptom level at the 3-year follow-up.

**Sleep variables**	**Some depressive symptoms** **OR (95% CI)**	***P*-value**	**Depressed symptoms** **OR (95% CI)**	***P*-value**
**PSQI total score**				
Base model	1.09 (1.03–1.15)	**0.003**	1.09 (0.98–1.21)	0.115
Multivariable adjusted	1.07 (1.00–1.14)	**0.036**	1.03 (0.97–1.16)	0.669
**PSQI >5 PSQI ≤5**				
Base model	1.44 (1.03–2.02)	**0.032**	2.03 (1.09–3.82)	**0.026**
Multivariable adjusted	1.36 (0.94–1.96)	0.104	1.84 (0.94–3.66)	0.077
**Sleep quality factor**				
Base model	1.18 (0.88–1.56)	0.266	1.22 (0.71–2.09)	0.467
Multivariable adjusted	1.10 (0.76–1.59)	0.601	0.71 (0.35–1.40)	0.337
**Sleep latency factor**				
Base model	1.25 (1.03–1.51)	**0.022**	1.31 (0.91–1.85)	0.132
Multivariable adjusted	1.15 (0.94–1.41)	0.173	1.32 (0.89–1.94)	0.16
**Sleep duration factor**				
Base model	1.16 (0.91–1.46)	0.221	1.05 (0.65–1.62)	0.822
Multivariable adjusted	1.10 (0.86–1.40)	0.455	0.90 (0.52–1.48)	0.705
**Sleep efficiency factor**				
Base model	1.25 (1.09–1.44)	**0.002**	1.34 (1.04–1.72)	**0.024**
Multivariable adjusted	1.21 (1.04–1.41)	0.014	1.21 (0.91–1.61)	0.173
**Sleep disturbances factor**				
Base model	1.44 (1.00–2.07)	**0.0496**	1.03 (0.48–2.08)	0.939
Multivariable adjusted	1.29 (0.88–1.89)	0.186	0.76 (0.33–1.65)	0.5
**Sleep medication use factor**				
Base model	1.58 (0.69–4.35)	0.286	1.46 (0.12–5.52)	0.605
Multivariable adjusted	1.14 (0.43–3.21)	0.776	1.27 (0.11–5.16)	0.741
**Daytime dysfunction factor**				
Base model	1.08 (0.88–1.31)	0.471	0.98 (0.64–1.43)	0.931
Multivariable adjusted	1.06 (0.86–1.32)	0.575	0.85 (0.52–1.31)	0.497

*GDS, Geriatric Depression Scale; PSQI, Pittsburgh Sleep Quality Index; CI, confidence interval. The base model was not adjusted, and the multivariable model was adjusted for age, sex, education, marriage, drinking, smoking, and self-reported health status. Bold means statistically significant*.

### Associations Between Sleep Disturbances and Worsening of Depressive Symptoms at Follow-Up

Table 4 shows that ~38.7% (*n* = 263) of older Chinese adults showed a ≥ 2-point increase in depressive symptoms. However, we observed no significant association between baseline PSQI total score or PSQI subcomponent scores and worsening of depressive symptoms at follow-up.

**Table 4:** Associations between baseline sleep disturbance and odds of increased depressive symptoms at the 3-year follow-up.

**Sleep factors**	**≥2 Point increase in GDS** **OR (95% CI)**	***P*-value**
**PSQI Total Score**		
Base model	1.05 (0.99–1.10)	0.086
Multivariable adjusted	1.03 (0.97–1.08)	0.359
**PSQI >5 PSQI ≤5**		
Base model	1.30 (0.95–1.79)	0.102
Multivariable adjusted	1.14 (0.82–1.59)	0.344
**Sleep quality factor**		
Base model	1.14 (0.88–1.49)	0.313
Multivariable adjusted	1.12 (0.81–1.57)	0.487
**Sleep latency factor**		
Base model	0.96 (0.80–1.15)	0.683
Multivariable adjusted	0.95 (0.78–1.14)	0.563
**Sleep duration factor**		
Base model	1.09 (0.87–1.36)	0.445
Multivariable adjusted	1.03 (0.82–1.31)	0.774
**Sleep efficiency factor**		
Base model	1.09 (0.95–1.24)	0.216
Multivariable adjusted	1.09 (0.94–1.25)	0.254
**Sleep disturbances factor**		
Base model	0.98 (0.69–1.39)	0.927
Multivariable adjusted	0.97 (0.67–1.39)	0.857
**Sleep medication use factor**		
Base model	1.33 (0.58–3.32)	0.482
Multivariable adjusted	1.13 (0.41–2.84)	0.788
**Daytime dysfunction factor**		
Base model	1.12 (0.93–1.35)	0.234
Multivariable adjusted	1.10 (0.90–1.35)	0.358

*Odds ratios and 95% confidence intervals for worsening depressive symptoms (≥2-point increase in GDS score) are given for older adults with sleep disturbances at baseline compared to those without sleep disturbances. Base models were crude models without adjusting any variables. Multivariable models were adjusted for age, sex, education, marriage status, smoking status, drinking status and self-reported health status. CI, confidence interval; GDS, Geriatric Depression Scale; PSQI, Pittsburgh Sleep Quality Index*.

## Discussion

In this longitudinal analysis of Chinese community-dwelling older people with few or no depressive symptoms at baseline, poorer global sleep quality, and sleep efficiency there appeared to be risk factors for “some depressive symptoms” but not increased risk of worsening of depressive symptoms at follow-up (~3.5 years later). In the cross-sectional analysis, we observed that the level of depressive symptoms was associated with sleep disturbances (self-reported poor global sleep quality and sleep efficiency). Overall, the cross-sectional association between sleep disturbances and depressive symptoms were attenuated in our longitudinal analysis. To our knowledge, we are the first to investigate whether baseline sleep disturbances could increase the risk of worsening depressive symptoms at follow-up in an older Chinese population. More importantly, we found for the first time that those with more reported sleep disturbance at baseline had greater odds of developing “some depressive symptoms.”

One cross-sectional study reported that sleep quality was a risk factor for depression, and the ORs of “depressive symptoms” and “some depressive symptoms” were 3.7- and 2.1-fold, respectively, for those with sleep disturbances at baseline (19). Another study also found that women with “some depressive symptoms” and “depressive symptoms” had greater odds of reporting poor sleep (20). In our cross-sectional analysis, we observed that the ORs for “depressive symptoms” and “some depressive symptoms” were 3.56- and 3.81-fold, respectively, for those with sleep disturbances at baseline which were consistent with the above two studies. A longitudinal study in a large cohort of older men revealed associations between worse sleep quality at baseline and more depressive symptoms at follow-up ~3.4-years later (8). A previous longitudinal study in a large cohort reported that older women with few or no depressive symptoms at baseline who were reported to have sleep disturbances had a greater risk of worse depressive symptoms 5 years later (7). Interestingly, our longitudinal analysis indicated that among non-depressed older adults at baseline, poor self-reported global sleep quality was associated with “some depressive symptoms” but not the risk of worsening depressive symptoms. This discrepancy could have been due to the shorter follow-up period (~3.5 years) in the current study. Another possible reason is that the SOF included only female participants and the MrOS included only male participants. Hence, these findings require verification in more cohorts.

In our cross-sectional analysis, we observed that sleep efficiency and daytime sleep PSQI subcomponents were associated with depressive symptoms regardless of adjustment for confounding factors. In the longitudinal analysis, the sleep efficiency PSQI subcomponent at baseline was associated with “some depressive symptoms” at the 3.5-year follow-up after adjusting for confounding factors. This interesting observation indicated that in older adults without depression, poor sleep efficiency may increase the risk of “some depressive symptoms.” On the other hand, poor sleep efficiency had no association with “depressive symptoms” at the 3.5-year follow-up after adjusting for confounding factors. The studies mentioned above (7, 8) did not find any relationship between sleep efficiency and depression. This discrepancy could be due to the differences in age, as the latter study consisted of women only, most of whom were older than 80 years, whereas the current study included men and women who were mostly younger than 80 years. A previous study reported that older adults show decreased sleep efficiency over time, with an 18.6% decline observed between 40 and 100 years of age (21). In addition, we found that the daytime sleep PSQI subcomponents were associated with depressive symptoms. This observation was consistent with previous population-based studies, which have examined the association between daytime sleep and depressive symptoms (22). We detected no significant association between PSQI scores at baseline and the odds of worsening depressive symptoms (≥2 point increase in GDS). The main difference could be due to the differences in population and sample size.

Marta Jackowska et al. reported that compared to an optimal duration, short (≤ 5 h) but not long (≥8 h) sleep hours were linked to elevated depressive symptoms (23). Sun et al. indicated that short sleep duration (<5 h, 5–6 h) significantly impacted depressive symptoms, while long sleep duration (>9 h) had no association with depressive symptoms (24). Lai et al. found that long sleep duration (≥ 9 h) was a risk factor for depression (22). Our study did not establish a relationship between sleep duration and the worsening of depressive symptoms. This discrepancy may be because the English Longitudinal Study of Aging (ELSA) included an English cohort, while the China Health and Retirement Longitudinal Study (CHARLS), Yilan Study in Taiwan (YILAN) and RuLAS included Chinese cohorts. Alternatively, the differences may have been influenced by time cutoffs, as Marta Jackowska took 7–8 h as a reference, Sun et al. took 7–8 h as a reference, and Lai et al. took 6–7 h as a reference. In addition, various studies have used different sleep and depression scales. The ELSA used the 8-item Centre for Epidemiological Studies Depression scale (CES-D), the CHARLS used the 10-item version of the Centre for Epidemiological Studies Depression scale (CESD-10), and the YILAN cohort used the Hospital Anxiety and Depression Scale (HADS). Hence, further investigation requires verification in other cohorts.

In summary, the current study assessed global sleep quality among older adults using the PSQI and validated the relationship between baseline sleep disturbances and follow-up depressive symptoms. However, there are several limitations of this analysis. First, our analysis was designed to make use of data that were collected as part of a large study. Hence, it was not designed to address our hypothesis, and the outcome measures were not predefined. Second, depressive symptoms were assessed by questionnaire rather than standard criteria for depression such as those from the International Classification of Diseases (ICD) or Diagnostic and Statistical Manual of Mental Disorder (DSM). Therefore, conclusions about psychiatric diagnosis cannot be made with certainty. Third, factors such as personal medical issues, antidepressant use, and family stress that may impact both sleep and mental health outcomes were not accounted for in this study. Finally, the generalizability of the study was limited, and more studies are needed to validate these findings in other cohorts.

In conclusion, findings of the present study contribute to the current literature in terms of the relationship between sleep disturbance and depressive symptoms in an older Chinese population. Together with the observations in the aforementioned studies, poor global sleep quality and sleep efficiency may be risk factors for depression and can predict levels of depressive symptoms.

## Data Availability Statement

The original contributions presented in the study are included in the article/supplementary material, further inquiries can be directed to the corresponding author/s.

## Ethics Statement

The Human Ethics Committee of the School of Life Sciences of Fudan University, Shanghai, China, approved this research (No: BE1815). Written consent was obtained from all participants prior to participation.

## Author Contributions

CQ: conceptualization, data analysis, and writing-original draft preparation. YY and XiaW: conceptualization and writing-reviewing and editing. HZ, QS, and XiW: data collection and data cleaning. All authors contributed to the article and approved the submitted version.

## Funding

This work was supported by the Shanghai Municipal Science and Technology Major Project #1 under Grant No. 2017SHZDZX01 and National Key R&D Program of China #2 under Grant No. 2018YFC2000400 and 2018YFC2000400-3.

## Conflict of Interest

The authors declare that the research was conducted in the absence of any commercial or financial relationships that could be construed as a potential conflict of interest.

## Publisher's Note

All claims expressed in this article are solely those of the authors and do not necessarily represent those of their affiliated organizations, or those of the publisher, the editors and the reviewers. Any product that may be evaluated in this article, or claim that may be made by its manufacturer, is not guaranteed or endorsed by the publisher.
